# Fabrication and Evaluation of Porous dECM/PCL Scaffolds for Bone Tissue Engineering

**DOI:** 10.3390/jfb14070343

**Published:** 2023-06-29

**Authors:** Weiwei Wang, Xiaqing Zhou, Zhuozhuo Yin, Xiaojun Yu

**Affiliations:** Department of Biomedical Engineering, Charles V. Schaefer School of Engineering and Sciences, Stevens Institute of Technology, Hoboken, NJ 07030, USA; wwang19@stevens.edu (W.W.); xzhou12@stevens.edu (X.Z.); zyin12@stevens.edu (Z.Y.)

**Keywords:** PCL materials, decellularized ECM, porous scaffold with anisotropic channels, mineral deposition, bone regeneration

## Abstract

Porous scaffolds play a crucial role in bone tissue regeneration and have been extensively investigated in this field. By incorporating a decellularized extracellular matrix (dECM) onto tissue-engineered scaffolds, bone regeneration can be enhanced by replicating the molecular complexity of native bone tissue. However, the exploration of porous scaffolds with anisotropic channels and the effects of dECM on these scaffolds for bone cells and mineral deposition remains limited. To address this gap, we developed a porous polycaprolactone (PCL) scaffold with anisotropic channels and functionalized it with dECM to capture the critical physicochemical properties of native bone tissue, promoting osteoblast cells’ proliferation, differentiation, biomineralization, and osteogenesis. Our results demonstrated the successful fabrication of porous dECM/PCL scaffolds with multiple channel sizes for bone regeneration. The incorporation of 100 μm grid-based channels facilitated improved nutrient and oxygen infiltration, while the porous structure created using 30 mg/mL of sodium chloride significantly enhanced the cells’ attachment and proliferation. Notably, the mechanical properties of the scaffolds closely resembled those of human bone tissue. Furthermore, compared with pure PCL scaffolds, the presence of dECM on the scaffolds substantially enhanced the proliferation and differentiation of bone marrow stem cells. Moreover, dECM significantly increased mineral deposition on the scaffold. Overall, the dECM/PCL scaffold holds significant potential as an alternative bone graft substitute for repairing bone injuries.

## 1. Introduction

Repairing and regenerating large bone defects remain significant challenges in the clinic. Although autografts have been considered the “gold standard” for repairing bone, several drawbacks have limited their use, including additional surgical procedures, increased risk of infection, increased blood loss, limited quantity, and the hypersensitivity or morbidity of the donor site [1]. Allografts cause patients less pain during the initial recovery and no donor site morbidity. However, they may have a slower rate of graft incorporation and a higher chance of disease transmission [2,3,4]. Similar to allografts, xenografts have the potential for disease transmission. They also involve issues of immune responses [5,6].

In recognition of the limitations of the bone graft methods described above, more and more attention has been paid to tissue-engineered bone scaffolds [7,8], which are three-dimensional (3D) matrices that allow and stimulate the attachment and proliferation of osteoinductive cells on their surfaces. During tissue formation, tissue-engineered scaffolds provide cells with a temporary platform to attach, proliferate, and differentiate. They also define an influential microenvironment that regulates cellular responses and subsequently affects the synthesis of the new tissue matrix for various functions [9]. Researchers have investigated multiple materials such as polycaprolactone (PCL) [10,11,12,13,14], poly(lactide-co-glycolic acid) (PLGA) [15,16,17,18], tri-calcium phosphate (β-TCP) [19,20], and hydroxyapatite [21,22,23,24,25] to fabricate such 3D bone scaffolds. Among all the different materials, PCL was commonly used in the fabrication of bone scaffolds due to its high mechanical strength, low melting and glass transition temperatures, and high thermal stability.

Ideally, 3D scaffolds should maximally replicate the natural growing environment of the cells to induce the desirable expression of cell phenotypes and tissue matrix synthesis [26]. However, due to the limitations of the fabrication technology and the limited available knowledge of the natural cell-growing environment, minimal progress has yet to be made toward the development of biomimetic 3D scaffolds [27,28,29]. Some researchers have tried incorporating biomolecules such as collagen, chitosan, and fibrinogen into scaffolds. Some groups have used biomolecules as a surface coating, while others have tried blending biomolecules into scaffolds to make slow-release synthetic materials. Despite the cell affiliation that was improved by such incorporations, considering that the compositional and structural complexity of the native tissue matrix varies from tissue to tissue, the simple incorporation of such molecules in 3D scaffolds cannot fully recapture the uniqueness of each tissue matrix.

ECM is composed of various types of proteins and carbohydrates, which are assembled to form unique architectures that can interact with cells via cell surface receptors [30,31]. It also plays a pivotal role in regulating cell behaviors [32]. For example, in osteogenesis, ECM is involved in cells’ adhesion, survival, proliferation, responses to growth factors, and differentiation, and, ultimately, the functional characteristics of the mature bone [33,34]. Therefore, the decellularized ECM (dECM) method was developed to provide people with a better way to mimic the native ECM closely. This method uses scaffolds by removing the cellular components from the cells or ECM constructs [35,36,37,38]. The biggest advantage of dECM scaffolds over other methods is that they retain the components of the natural cell environment; with proper decellularization, the complex biomolecular and physical cues in the ECM are preserved and can support the cells’ growth and viability.

**Scheme 1:**
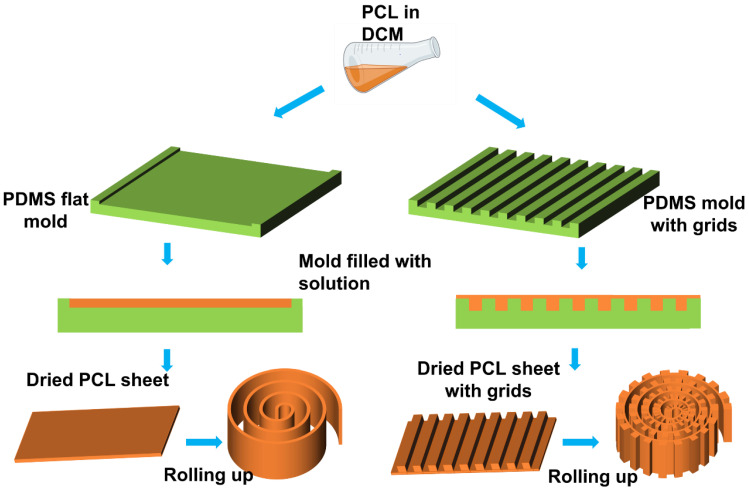
Schematic diagram of the step-by-step preparation of different scaffolds.

As well as the chemical composition, the scaffolds’ architecture and the surface topography of scaffolds regulate the cells’ behaviors [39]. Our group has designed a spiral-structured scaffold (see Scheme 1) that mimics the natural bone structure and provides open gaps, sufficient space, and thinner walls for cells’ attachment, migration, proliferation, and differentiation during in vitro culture [40,41]. Moreover, we also made the scaffolds porous through the salt leaching method. Compared with conventional cylindrical scaffolds, the porous spiral scaffold can provide better nutrient supply, oxygen infiltration, and waste removal [42]. In order to better mimic the native bone structures, anisotropic channels were introduced into PCL scaffolds and investigated for increasing their porosity and providing contact guidance for the osteoblasts to deposit the matrix in the axial direction for enhancing bone regeneration.

In this study, PCL/dECM scaffolds were prepared in spiral structures with different anisotropic channel sizes and porosities. The morphology and mechanical strength of the scaffolds were characterized. The scaffolds were further functionalized with dECM. The fibroblasts, endothelial cells, osteoblasts, and chondrocytes were cultured on PCL scaffolds, decellularized, and characterized. In order to investigate their in vitro osteogenesis capability, the cellular responses of rat bone marrow mesenchymal cells (rBMSC) seeded onto the scaffolds were studied. The effects of dECM on the scaffolds in terms of mineral deposition were also investigated.

## 2. Material and Methods

### 2.1. Materials

Unless otherwise indicated, all chemicals used for preparation of the scaffold and cell sheet decellularization were purchased from Sigma-Aldrich (St. Louis, MO, USA). Dichloromethane (DCM) was purchased from VWR Chemicals (Radnor, PA, USA).

All cells used were purchased from ATCC, except that the mesenchymal stem cells (MSC) were purchased from Innoprot (Bizkaia, Spain), and the human chondrocyte cells were purchased from Cell Application INC (San Diego, CA, USA). Culture reagents, including fetal bovine serum (FBS), penicillin–streptomycin, and trypsin, were purchased from Life Technologies (Carlsbad, CA, USA). The culture media were purchased from the following sources: rat mesenchymal basal medium and its growth supplement, Cell Application Inc.; Eagle’s minimum essential medium, ATCC; Dulbecco’s modified Eagle medium/nutrient mixture F-12, Gibco (Billings, MT, USA); Dulbecco’s modified Eagle medium, Gibco.

The analytical reagents were purchased from the following sources: CellTiter Blue cell viability assay, Promega (Madison, WI, USA); Quant-iT™ PicoGreen^®^ dsDNA, Invitrogen (Waltham, MA, USA); BCA assay kit, Thermo Fisher Scientific (Waltham, MA, USA).

### 2.2. PCL Scaffold Fabrication

To create channels within the scaffolds, 12% (*w*/*v*) polycaprolactone (PCL, Mw = 80,000 Sigma-Aldrich) was dissolved in DCM and poured onto premade polydimethylsiloxane (PDMS) molds with 100 μm and 500 μm grids. In order to make porous scaffolds, sodium chloride was added to the PCL solution at a concentration of 30 mg/mL. Next, two sieves with a mesh size of 0.074 mm and 0.100 mm excluded salt particles. After the PCL solution solidified to form a thin sheet, the PDMS molds were removed to create the inner channels. Salt particles were then removed by placing the PCL sheets in the mixture of isopropyl and chloroform for 30 s then they were transferred to water and stirred overnight. The resulting porous PCL sheets were then dried in the oven at 40 °C for another 24 h. To make the spiral structured scaffolds, around 10 layers of PCL sheets were stacked together and wrapped around a 21 G needle, and the two ends of the porous polymeric film were then sealed with a small amount of DCM to make a tubular scaffold with a length of 5 mm, an outer diameter of 5 mm, an inner diameter of 0.84 mm, and a thickness of 2.08 mm.

### 2.3. Test of the Mechanical Properties 

Disc-shaped samples (approximately 10 mm in diameter and 5 mm in height) were compressed at a rate of 15 mm/min using the Instron Model 5965 Material Test System. The modulus of different samples was calculated based on the resulting linear part of the stress–strain curves.

### 2.4. Porosity Test

The porosity of different scaffolds was tested using the water intrusion method. Samples were submerged in distilled de-aired water (DDW) and put into a negative-pressure cabinet for 24 h. They were then taken out and stood on a petri dish to remove excess water. The change in mass before and after soaking in DDW was measured with an analytical balance. This change in mass was the mass of water trapped in the pores. The percentage was calculated by the equation below

(W_after_ − W_before_)/ρ_water_/V∗100%.

where W_after_ is the dry mass of samples, W_before_ is the mass of the sample saturated with water, and V is the sample’s volume calculated from the vernier caliper’s measurements.

### 2.5. Cell Culture

Osteoblast hFOB cells were cultured in DMEM/F12 medium supplemented with 10% FBS and 1% P/S. Human dermal fibroblast cells were cultured in an EMEM medium supplemented with 15% FBS and 1% P/S. Rat MSC cells were cultured in rat mesenchymal basal medium with a growth supplement. Chondrocyte cells and EA.hy926 endothelial cells were cultured in DMEM medium supplemented with 10% FBS and 1% P/S. All cells were dynamically seeded onto porous PCL scaffolds using a spinner flask containing 50 mL of the culture medium. The seeding density was around 1 × 10^6^ cells per flask, and the spinning speed was set at 35 rpm. Cell attachment was examined by methylene blue staining. Cell proliferation on different scaffolds was investigated by the CellTiter Blue assay kit.

### 2.6. Decellularization

PCL scaffolds with fibroblasts, endothelial cells, osteoblasts, and chondrocytes were decellularized according to a previously published protocol [43,44]. Briefly, the scaffolds were washed thrice with PBS and placed in a 10 mM phosphate-buffered 50 mM sodium solution containing 125 mM 3-(decyl-dimethyammonio) propane sulfonate (SB3-10) overnight at 4 °C under gentle agitation. After another three PBS washes, the scaffolds were placed in a 50 mM sodium solution buffered with 10 mM phosphate containing 0.14% sodium deoxycholate and 0.6 mM 3-(N, N-Dimethyl-palmitylammonio)propanesulfonate (SB3-16) overnight at 4 °C under gentle agitation. This process was repeated twice to ensure the removal of cell debris. Decellularized scaffolds were stored in PBS containing a protease inhibitor cocktail at 4 °C.

### 2.7. Evaluation of Decellularization Based on Protein and DNA Content

The BCA protein assay and the Quant-iT PicoGreen dsDNA assay were used to quantify the protein and DNA content.

Each decellularized sample was soaked in a RIPA buffer with a proteinase inhibitor and a phosphatase inhibitor and vortexed for 30 min at 4 °C to extract all the protein. The mixture containing the protein extract and other solid debris was centrifuged at 12,000 rpm for 20 min at 4 °C. The supernatant was collected, and the protein content was quantified by a BCA assay according to the manufacturer’s protocol.

For DNA extraction, dECM samples were soaked in a TE buffer, and the thermal shock method was used to break any possible remaining cells. The samples were then submerged in a mixture of 20% SDS and Triton-100 at a ratio of 4:6 for purification. The resulting DNA content after recovery was examined according to the manufacturer’s protocol.

Both results were read using a BioTek Synergy HT plate reader.

### 2.8. Cell Differentiation on Scaffolds

Two differentiation markers of bone marrow mesenchymal stem cells, osteopontin and Type I collagen (COL-1), were used to examine the process of osteogenesis. Briefly, after 7, 14, 21, and 28 days of incubation, the scaffolds were washed with cold PBS three times, fixed in 1 mL of a 4% formaldehyde solution for 10 min, and permeabilized in 1 mL of 0.5% Triton X-100 for 10 min at room temperature. The scaffolds were then blocked in 1 mL of a solution containing 3% BSA for 30 min. Afterward, the samples were incubated with the osteopontin (Abcam, Boston, MA ab8448, from rabbits) or Collagen Type I (Abcam, ab292, from rabbits) antibodies for 1 h at room temperature and subsequently visualized with fluorescein (FITC)-conjugated AffiniPure goat anti-rabbit IgG(H+L). Next, the scaffolds were immersed in a phalloidin (conjugated with Alexa Fluor 488) solution for 45 min to show the cell skeleton, and then the scaffolds were rinsed with PBS and stained for 10 min with 4′,6-diamidino-2-phenylindole (DAPI) to label the nuclei. Finally, the scaffolds were visualized under a fluorescence microscope.

### 2.9. ALP Assay

At predesignated time intervals of 7, 14, 21, and 28 days, the phenotype differentiation of the osteoblasts was measured using the ALP assay kit according to the manufacturer’s instructions. In detail, the scaffolds were taken out and washed twice with PBS. Each scaffold (n = 3) was then transferred to a 1.5 mL Eppendorf tube containing 1 mL of the assay buffer. The samples were centrifuged at 15,000 rpm/min for 15 min at 4 °C to remove any insoluble materials. Next, the supernatant was collected and transferred to a new Eppendorf tube and 50 μL of a 5 mM *p*NPP solution was added to each well (96-well plate) containing 80 μL of the supernatant above. Afterward, the 96-well plate was incubated at room temperature for about 60 min in the dark, and, subsequently, 20 μL of a stop solution was added to stop the reaction. At the same time, a fresh set of standards were also prepared. Specifically, 10 μL of the ALP enzyme solution was added to 120 μL of each *p*NPP standard well and incubated for 1 h, followed by adding 20 μL of the stop solution. The absorbance was measured at 405 nm using a UV-vis microplate reader, and the ALP activity (U/mL) was calculated as follows

ALP activity (U/mL) = A/V/T ∗ f

where A is the amount of pNPP generated by the samples (in μmol), V is the volume of the sample added in the assay well (in mL), T is the reaction time (in min), and f is the sample’s dilution factor.

In this study, the reaction time was set at 60 min, the original sample volume was 80 μL, and the dilution factor was 1.

### 2.10. Biodeposition

Scaffolds with/without deposited dECM were soaked overnight in 30% (*v*/*w*) bovine serum albumin (BSA) and washed with deionized (DI) water three times to remove any extra BSA. Simulated body fluid (SBF) was prepared according to Kokubo’s recipe [45]. The scaffolds were then incubated in a solution of SBF and maintained at 37 °C for mineral deposition. The SBF was renewed every 24 h. After being incubated for predetermined time periods (7, 14, and 21 days), the samples were removed from the solution and immersed in DI water overnight to remove soluble inorganic ions. The samples were then stained with Alizarin Red S (ARS) staining solution to check the deposition of calcium, followed by documenting the images. The quantification of ARS was carried out by cetylpyridinium chloride (CPC) extraction. Briefly, scaffolds with ARS were incubated in a 10% CPC solution for 30 min with agitation to fully extract the ARS. The extraction was then read with a BioTek Synergy HT plate reader at A_562_ together with several standard gradient solutions of ARS and CPC.

### 2.11. Morphological Characterization

#### 2.11.1. Scanning Electron Microscopy

The morphology of the prepared microspheres was examined by SEM (Zeiss Auriga FIB-SEM with a Leica VCT-100 cryo-system, Oberkochen, Germany). Briefly, the samples with dECM were dried with increasing ethanol concentrations (50, 70, 80, 90, 95, and 100%); the other samples were air-dried. All samples were attached to the scanning electron microscope’s stages with double-sided adhesive tape, then sputter-coated with gold for 1 min using a Denton Desk-1 sputter coater. THE surfaces were visualized at an accelerating voltage of 3 kV using an AMray 1830-D4 equipped tungsten gun.

At least five randomly selected SEM images were acquired for each type of scaffold. The images were manually measured and analyzed using ImageJ software v1.5 (NIH, Bethesda, MD, US) to calculate the average microsphere diameter and pore size.

#### 2.11.2. Stereomicroscopy

Cell-laden scaffolds stained with methylene blue, PCL sheets, and scaffolds stained with Alizarin Red were examined with a Nikon SMZ1500 stereomicroscope with a DS-Fi1 digital camera.

#### 2.11.3. Fluorescent Microscopy

Immunofluorescent stained scaffolds were examined with a Nikon Eclipse 80i epifluorescent microscope and a Zeiss confocal microscope.

### 2.12. Statistical Analysis

All the data above were presented as the mean ± standard deviation and evaluated by the one-way ANOVA test. The results were considered statistically significant at a *p*-value less than 0.05.

## 3. Results and Discussion

### 3.1. Fabrication of the PCL Scaffolds 

**Figure 1:**
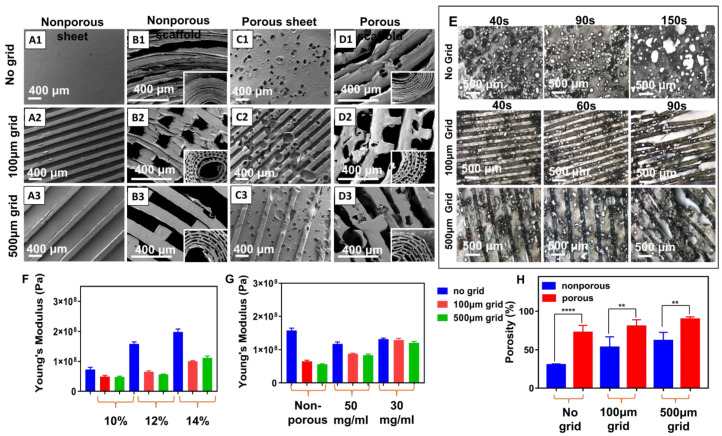
(**A1**–**A3**) SEM images of PCL sheets with different grid sizes: 0 μm, 100 μm, and 500 μm. (**B1**–**B3**) SEM images of hard-shell scaffolds made from different PCL sheets. From top to bottom: sheets with 0 μm, 100 μm, and 500 μm grids. (**C1**–**C3**) SEM images of porous PCL sheets with different grid sizes: 0 μm, 100 μm, and 500 μm. (**D1**–**D3**) SEM images of the hard-shell scaffold made from different porous PCL sheets. From top to bottom: sheets with 0 μm, 100 μm, and 500 μm grids. (**E**) Images of different PCL sheets soaked in an IPA/chloroform mixture for different time periods. (**F**) Linear part of the stress–strain curves of nonporous scaffolds made from different PCL concentrations. (**G**) Linear part of the stress–strain curves of porous scaffolds made with different NaCl concentrations. (**C**,**D**) Analysis of Young’s modulus of different scaffolds. (**H**) Porosity of different scaffolds. (**, *p* ≤ 0.01; ****, *p* ≤ 0.0001).

The process of fabricating different PCL scaffolds is shown in Scheme 1. In order to determine the most suitable channel size for the PCL scaffolds, three groups of PCL sheets were prepared (Figure 1A1–A3): a flat sheet (no grid), a 100 μm grid, and a 500 μm grid. The scaffolds made from flat sheets had minimal space between the different layers, which led to higher mechanical properties but poor cell affinity (Figure 1B1). On the contrary, scaffolds made from PCL sheets with 500 μm grids left multiple large channels for oxygen and nutrient exchange. Consequently, they were predicted to lead to better cell attachment and viability (Figure 1B3). However, the hollow space/porous structure weakened the mechanical properties of the scaffolds. Scaffolds with 100 μm grids also provided channels for oxygen and nutrient exchange. Still, the smaller channel size would sacrifice fewer mechanical properties, such as Young’s modulus, compared with scaffolds with 500 μm grids. Therefore, 100 μm was selected as the channel size for our scaffolds.

To further encourage the cells’ attachment to and growth on the scaffolds, the salt leaching method was used to fabricate porous PCL sheets. Several grain sizes of sodium chloride were studied for making porous PCL sheets. The grains with a large size (>100 μm) could hardly be distributed evenly on the PCL sheets; the aggregation would make huge pores on the sheet and therefore tear the sheet apart. On the other side, grains with too small a size (<50 μm) were easy to trap in the PCL sheets and difficult to expose to the outside. Even with the grain size currently used (75–100 μm), water washing alone was not enough to release all the sodium chloride from the sheet, since there was a thin layer of PCL film that blocked the salt from contacting the water. The mixture of isopropyl alcohol and chloroform at a ratio of 19:1 was used to fully release the sodium chloride because it could mildly dissolve the PCL encapsulated around the salt granules. The soaking times of different PCL sheets in the mixture differed as the thickness of the thinnest part in the sheets differed (Figure 1D1–D3). The thickness of the PCL sheets with no grid was even all over the material, around 50 μm. Thus, the soaking time would be longer than for sheets with grids. For the no-grids group, the proper soaking time was 90 s because 40 s of soaking could only open partial encapsulations, while 150 s of soaking tore the sheets. For gridded groups, no matter what the grid size was, the thinnest part was around 20 μm (Figure 1D2,D3). Therefore, the proper soaking time was 60 s.

### 3.2. Mechanical Tests

As all PCL sheets were finally rolled up into tubular scaffolds, the softness of all the sheets was restricted to a specific hardness range. Several parameters were studied within this range: the concentration of PCL in the nonporous scaffolds (10%, 12%, and 14%), which also served as control groups, and the concentration of NaCl in the porous scaffolds (30 mg/mL and 50 mg/mL). The results of the mechanical test for different groups are shown in Figure 1F,G. According to the results for Young’s modulus, it can be seen that a larger grid size resulted in lower mechanical properties.

For the nonporous scaffolds, an increased PCL concentration caused greater hardness and, consequently, created more difficulty in terms of fabrication. The high concentration of PCL made the solution more viscous. As the DCM evaporated during fabrication of the sheet, the viscosity of the solution increased further, making the PCL sheets tend to show uneven thickness. However, the modulus of the 10% PCL no-grid scaffold was only 72.91 MPa, which was far from the actual modulus of bone. Typically, Young’s modulus of cortical bone is 17–20 GPa along the longitudinal axis and 6–13 GPa along the transverse axis [46]. The modulus of trabecular bone ranges between 10 and 3000 MPa [47]. Moreover, the nonporous scaffolds only served as control groups, and the porous scaffolds were the ultimate scaffolds used for bone generation. These porous structures would weaken the strength of the scaffold further under the same fabrication conditions. The no-grid scaffolds in both the 12% group and 14% group achieved a modulus greater than 150 MPa, but the modulus of the scaffolds with grids in these groups dropped significantly compared with the no-grid ones. This is because the increased hardness made the PCL sheets more challenging to roll up, and had a higher chance of having twisted grids inside the scaffold. Such chaos would end up in lower strength.

Each PCL sheet can encapsulate limited NaCl granules. According to the experimental results, the maximum concentration of NaCl was 50 mg/mL in the PCL solution. Higher NaCl concentrations would form an extra layer of salt on the porous sheet and sometimes cause aggregations. Therefore, adding more NaCl to the PCL solution barely helped improve the scaffold’s porosity. When the NaCl concentration decreased to 30 mg/mL, the stress–strain curves of all the scaffolds were surprisingly close to each other (Figure 1F), and even scaffolds with grids achieved a modulus greater than 120 MPa. It was predicted that when there is cell attachment and mineralization, the mechanical properties of the scaffold could be improved further.

### 3.3. The Porosity of Different PCL Scaffolds

The porosity test results are shown in Figure 1H. It can be seen that both the scaffolds made with nonporous sheets and the porous sheets had porosity. There were two reasons leading to porosity in the scaffolds made with nonporous PCL sheets. First, there were small gaps between each layer of the PCL sheets as they were handmade. Second, because there were many grids on the PCL sheets, when the sheets were rolled up into a column, the grids formed many small channels that could be considered to be pores. However, compared with the porous group, the porosity of all three nonporous groups was significantly lower. In both the nonporous and porous groups, the no-grid scaffolds had the lowest porosity. The nonporous no-grid scaffolds had an average porosity of 30.38%, and the average porosity of the porous no-grid scaffolds was 72.51%. The 500 μm scaffolds had the highest porosity in both groups. The porosity of the nonporous 500 μm scaffolds was 61.89%, whereas the porosity of the porous 500 μm scaffolds was 89.55%. The porosity of the porous groups was so high that even the group with the lowest porosity (the porous no-grid group) was 10% higher than the nonporous 500 μm group, which had the highest porosity among three groups made with nonporous PCL sheets. Furthermore, both the porous 100 μm and 500 μm groups had a porosity above 80%, indicating that they had a very large surface-to-volume ratio and, therefore, were more favorable for cell attachment and nutrient/oxygen infiltration.

### 3.4. Cell Attachment and Proliferation

**Figure 2:**
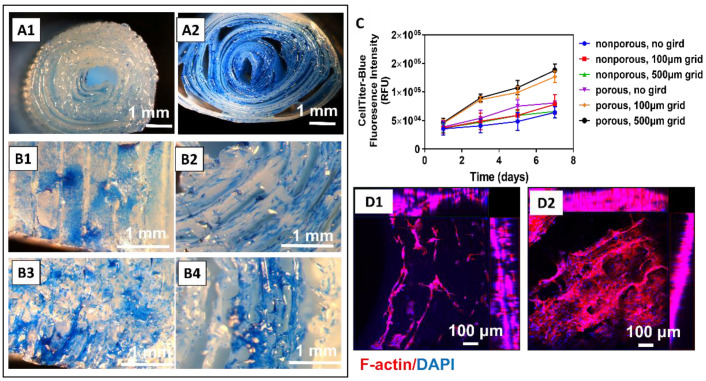
(**A1**) Methylene blue staining showing the attachment of hFOB cells on Day 3 under static seeding. (**A2**) Methylene blue staining showing the attachment of hFOB cells on Day 3 by dynamic seeding. (**B1**,**B2**) Methylene blue staining showing the attachment of hFOB cells on Day 3 on the nonporous scaffold with 500 μm grids. (**B3**,**B4**) Methylene blue staining showing the attachment of osteoblast cells on Day 3 on the porous scaffold with 500 μm grids. (**C**) CellTiter Blue results showing hFOB cells attached to the different scaffolds on Day 3, Day 5, and Day 7. (**D1**) Confocal image of the attachment of hFOB cells to the nonporous 100 μm scaffold on Day 7. (**D2**) Confocal image of the attachment of hFOB cells to the porous 100 μm scaffold on Day 7.

According to the results of the mechanical test, the scaffolds made from 12% PCL with and w/o 30 mg/mL NaCl were selected for the cell studies. Fibroblasts, endothelial cells, chondrocytes, and osteoblasts were seeded onto porous 500 μm grid scaffolds to test their proliferation. The results are shown in Figure S1. As the osteoblasts (hFOB) had the highest proliferation among all four cells, the results presented in this section are the results for hFOBs. As we can see from the methylene blue staining results, even with a seeding density of 200 k cells/scaffold, the scaffolds could obtain enough initial cell attachment from the static culture (Figure 2A1). The culture method was then changed to dynamic seeding in spinner flasks. Each flask contained 10 scaffolds, 50 mL of the culture medium, and 1 million cells. It is clear from Figure 2A2 that the attachment of cells to the scaffold was significantly improved compared with the static culture. Even in the no-grid scaffold, the space between two layers of the PCL sheets was covered by cells. Scaffolds with grids displayed the formation of a cell film (Figure 2B). The confocal images of the hFOB cells showed the difference in cell growth between the nonporous gridded scaffolds (Figure 2D1) and the porous gridded scaffolds (Figure 2D2) after 7 days of dynamic culture.

The proliferation rate of hFOB cells on the different scaffolds was tested using the CellTiter Blue assay kit. The results are shown in Figure 2C. Day 3’s results showed that porous scaffolds made with gridded PCL sheets obtained much higher cell attachment than other groups. The 100 μm grid and 500 μm grids seemed to have no difference in cell growth. However, by Day 7, the cell viability of the porous scaffolds with a 500 μm grid indicated that a larger channel size was still more beneficial for cell proliferation. The slope of the porous 500 μm grid group was the largest among all the curves.

### 3.5. Decellularization

**Figure 3:**
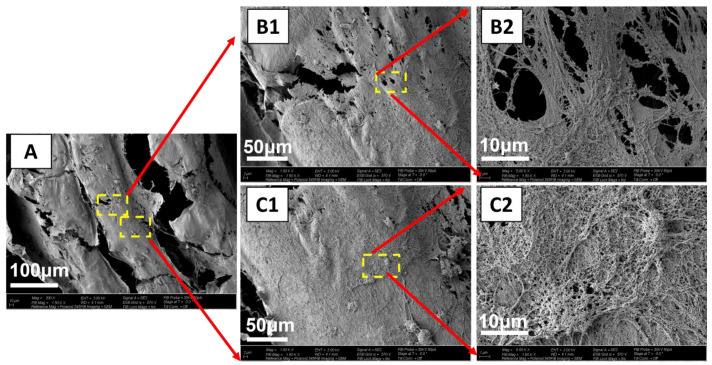
(**A**) SEM images of the dECM of the scaffold initially seeded with osteoblasts. (**B1**,**B2**) The dECM of the cells covered across the porous area of PCL scaffolds. (**C1**,**C2**) The dECM of cells attached to the nonporous area. Yellow boxes and red arrows indicated areas that were zoomed in.

Four types of cells were seeded onto a porous scaffold (made from 12% PCL and 30 mg/mL NaCl, with a 500 μm grid) for decellularization, namely osteoblasts (OB), fibroblasts (FB), endothelial cells (EC), and chondrocytes (CH). The cell-laden scaffolds were decellularized after 7 days of culture. The content of remaining DNA and of remaining protein was quantified by the picoGreen assay kit and the BCA protein assay, respectively. The results showed that the PCL-OB scaffolds had 6.09 ± 0.19 ng DNA/scaffold present, the PCL-FB scaffolds had 5.89 ± 0.18 ng DNA/scaffold, the PCL-EC scaffolds had 6.34 ± 0.17 ng DNA/scaffold, and the PCL-CH scaffolds had 6.29 ± 0.05 ng DNA/scaffold present. All four groups showed similarly low levels of DNA remaining, indicating that the decellularization process was very successful. On the other hand, the BCA results showed that protein contents had a different trend from DNA content. The PCL-FB scaffolds had 39.54 ± 6.08 μg protein/scaffold present, which was the highest protein content amount of the three groups. The PCL-OB scaffolds had 20.93 ± 4.01 μg protein/scaffold present, the PCL-EC scaffolds had 18.73 ± 5.00 μg protein/ present, and the PCL-CH scaffolds had 32.77 ± 4.53 μg protein/ scaffold present. Cell-material interactions brought the difference in protein amount across the four groups. Compared with other cell types, although endothelial cells proliferated fast, they tend to secrete the least of ECM. Therefore, this was the first cell type to be excluded from the following dECM studies. From the SEM images of the OB dECM scaffolds (Figure 3A), it was clear that after 7 days of cell culture, the ECM was able to cover the surface of the scaffold fully, no matter whether the area was porous or nonporous (Figure 3B1,B2,C1,C2). Moreover, the PCL-OB group had the second lowest amount of protein content left after decellularization, but the PCL scaffolds in this group still showed almost 100% coverage with ECM.

### 3.6. Reseeding Cells to dECM Scaffolds

**Figure 4:**
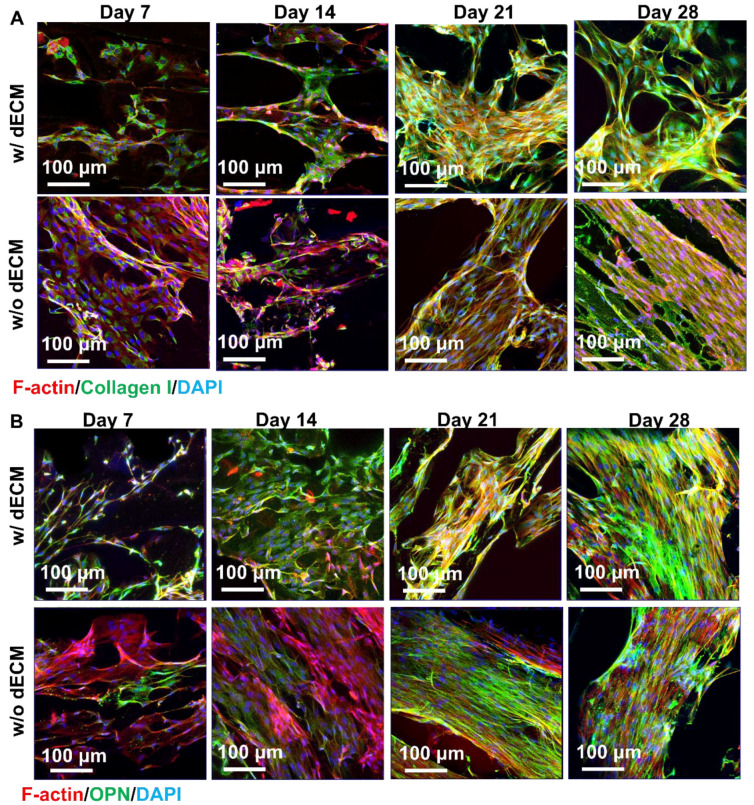
(**A**) Immunofluorescent images of Collagen I-stained rBMSC on the different scaffolds on Days 7, 14, 21, and 28. (**B**) Immunofluorescent images of osteopontin (OPN)-stained rBMSC on different scaffolds on Day 7, 14, 21, and 28.

Fibroblasts are abundant around the osteoblasts in bone tissue and accumulate massively at the beginning of bone regeneration. Moreover, the ECM secreted by fibroblasts has also been demonstrated to promote the of tissue regeneration, including muscles, nerves, arteries, and bone tissue [48,49]. The previous BCA results also indicated that fibroblasts secreted the most protein during the initial cell seeding. Therefore, a scaffold with fibroblast dECM (FB dECM) was picked for the following cell reseeding. The FB dECM scaffolds were soaked in an antibiotic–antimycotic solution for half an hour and washed five times with PBS. Rat mesenchymal stem cells (rBMSC) were then seeded dynamically to the dECM scaffolds at 50 k cell/scaffold concentrations. During the process of the rBMSC differentiating to osteoblasts, several markers indicated osteogenesis, such as Runt-related transcription factor 2 (Runx2), Collagen I, osteopontin, and osteocalcin. According to the steps in the osteogenic differentiation of MSCs, MSCs actively proliferate during the initial stages of osteogenesis and produce collagen. In the later stage of osteogenesis (14–28 days), there should be a high expression of osteopontin [50]. Consequently, Collagen I and osteopontin were chosen as the indicators of differentiation at different stages. The immunofluorescent images of osteopontin- and Collagen I-stained cells on scaffolds with and without dECM are shown in Figure 4. Compared with the pure PCL scaffold (w/o dECM), both Collagen I and osteopontin showed much higher and earlier expression in the dECM scaffold. The pure PCL group showed positive Collagen I staining from Day 14 and positive OPN staining from Day 21. At the same time, the dECM group showed positive signals from Days 7 and 14, respectively. This indicated that the PCL/dECM scaffolds promoted the differentiation of rBMSC better than the pure PCL scaffolds.

**Figure 5:**
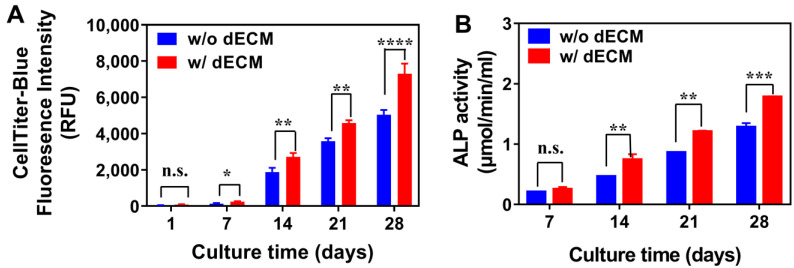
(**A**): CellTiter Blue assay showing rBMSC cells proliferating on scaffolds with and without dECM. (**B**) Alkaline phosphatase (ALP) activity secreted by rBMSC cells cultured on scaffolds with and without dECM. n.s., no significant difference; *, *p* ≤ 0.05; **, *p* ≤ 0.01; ***, *p* ≤ 0.005; ****, *p* ≤ 0.0001.

Not only the differentiation but also the proliferation of rBMSC cells on the dECM scaffolds showed a higher rate compared with the pure PCL scaffolds (see Figure 5A). From the results of the CellTiter Blue assay, it can be observed that on Day 1, both groups started with similar cell numbers. However, on Day 7, the dECM group began showing slightly greater cell proliferation; from Day 14 to Day 21, the difference between the two groups became more prominent. This is the evidence that the dECM could improve cell proliferation significantly.

Similar to the proliferation rate, the dECM group also showed better performance in terms of ALP activity. As shown in Figure 5B, no significant difference in ALP activity was observed between cells cultured on the PCL and PPBC scaffolds on Day 7. However, on Day 14, the ALP activity showed a substantial increase in the dECM scaffolds compared with the pure PCL scaffolds. Furthermore, the difference between the two groups increased as the culture time elongated. By Day 21, the difference between the two groups reached the maximum. On Day 28, the ALP activity of the dECM scaffolds decreased, meaning that the osteoblast cells in this group had finished differentiation. On the contrary, the ALP activity of the group without dECM still showed an increase, although the rate of increase became slower compared with the previous three weeks, indicating that the osteoblast cells in this group were still undergoing differentiation. In summary, these results implied that the incorporation of dECM could induce more cell growth and differentiation during the 28 day culture period compared with pure PCL.

### 3.7. Biodeposition

**Figure 6:**
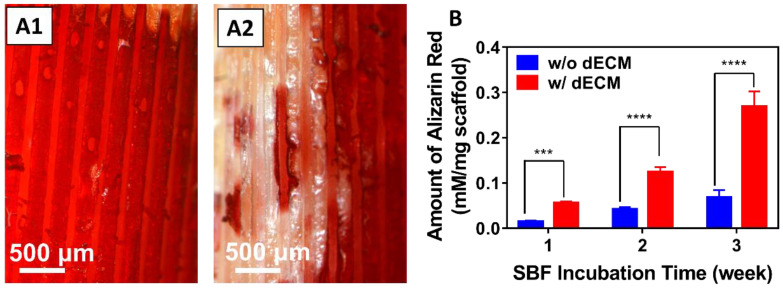
(**A1**) PCL/dECM scaffold stained with Alizarin Red on Day 28. (**A2**) Pure PCL scaffold stained with Alizarin Red on Day 28. (**B**) Quantification of Alizarin Red staining of samples with and without dECM at different time points. ***, *p* ≤ 0.005; ****, *p* ≤ 0.0001.

Simulated body fluid (SBF) was used to mimic in vivo mineral deposition on the scaffold during osteogenesis. Mineralization is the final step of new bone formation and will finally harden bone tissues [51]. Our previous work included a study to compare the biodeposition on nanofibers with and without a BSA coating, and with and without a dual BSA–dECM coating. The results showed that the dual coating on unmineralized surfaces proved to have a synergistic effect on osteoblasts’ growth and mineralization [52]. On the basis of these results, the BSA coating was used in this study to optimize the biodeposition. The results of Alizarin Red staining of the samples (with and w/o dECM) incubated in SBF for 3 weeks are shown in Figure 6A1,A2. Although the pure PCL without a dECM scaffold showed some area of positive ARS staining (Figure 6A2), the area of positive ARS staining in the dECM scaffold was much more extensive, and the ARS was more evenly distributed on the dECM scaffold as well (Figure 6A1). The quantification of Alizarin Red staining further confirmed that from the first week, the amount of biodeposition on the dECM scaffolds was about three times that of scaffolds w/o dECM at all time points.

## 4. Conclusions

In conclusion, we have successfully fabricated porous dECM/PCL scaffolds with multiple anisotropic channels for bone regeneration. The channels and pores on the scaffolds increased the porosity from 31% to over 80% and consequently increased cell attachment and nutrient/oxygen infiltration. The scaffolds made from 12% PCL, 30 mg/mL NaCl, and a 100 μm grid significantly improved cells’ proliferation and differentiation while maintaining Young’s modulus above 120 MPa. The dECM deposited on the scaffolds further enhanced bone marrow stem cells’ proliferation, future differentiation, and possible future osteogenesis. All biomarkers tested appeared at least one week earlier in the dECM scaffold compared with the pure PCL scaffold. Moreover, the dECM accelerated the process of mineral deposition onto the scaffolds. Therefore, the incorporation of dECM with the porous PCL scaffold with anisotropic channels could better recapture the critical physicochemical properties of native bone tissue and accelerate osteogenesis. The scaffold has the potential to be used as a bone graft substitute.

## Data Availability

Data are available upon request to the corresponding author.

## Supplementary Materials

The following supporting information can be downloaded at: https://www.mdpi.com/article/10.3390/jfb14070343/s1, Figure S1: CellTiter Blue results showing different cells attached and proliferated to porous 500 μm grid scaffolds on day 3, day 5, and day 7.
