# Supplementary material for: Fabrication and Evaluation of Porous dECM/PCL Scaffolds for Bone Tissue Engineering
Source: J Funct Biomater. 2023 Jun 29;14(7):343. doi: 10.3390/jfb14070343 (PMC10381742; doi:10.3390/jfb14070343)
Supplement: Supplementary file 1 [file jfb-14-00343-s001.zip › jfb-2438245-supplementary.pdf]

Supplementary Materials

Fabrication and Evaluation of Porous dECM/PCL Scaffolds for Bone Tissue Engineering

Weiwei Wang,[†] Xiaqing Zhou,[†] Zhuozhuo Yin,[†] Xiaojun Yu*,[†]

[†] Department of Biomedical Engineering, Charles V. Schaefer School of Engineering and Sciences, Stevens Institute of Technology, Hoboken, New Jersey, 07030, United States.

*Corresponding author:

Xiaojun Yu, PhD

Professor

Department of Biomedical Engineering, Charles V. Schaefer School of Engineering and Sciences, Stevens Institute of Technology,

Hoboken, New Jersey 07030, United States.

Tel.: +1-201-216-5256

E-mail address: xyu@stevens.edu

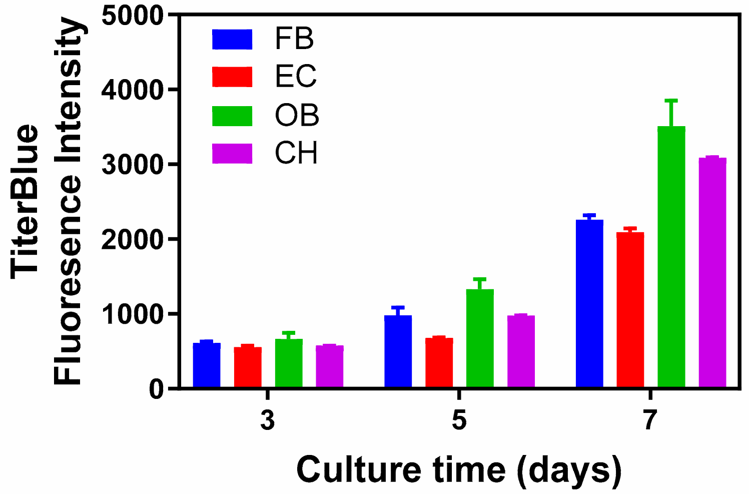

Figure S1. CellTiter Blue results showing different cells attached and proliferated to porous 500 μm grid scaffolds on day 3, day 5, and day 7.
